# The Impact of Polypill on Adherence and Cardiovascular Outcomes: A Comprehensive Systematic Review with Meta-Analysis

**DOI:** 10.2174/011573403X283174240110025442

**Published:** 2024-01-23

**Authors:** Hamza Salim, Basel Musmar, Motaz Saifi, Mohammed Ayyad, Mohammed Ruzieh, Jehad Azar, Zaher Nazzal

**Affiliations:** 1 Department of Medicine, An-Najah National University, Nablus, Palestine;; 2 Department of Cardiology, University of Florida, Gainesville, Florida, USA;; 3 Department of Medicine, Mayo Clinic, Phoenix, AZ, USA

**Keywords:** Polypill, cardiovascular, cardiovascular disease, hypertension, diastolic blood pressure, CVD risk factor

## Abstract

**Background:**

Cardiovascular disease (CVD) remains a leading cause of morbidity and mortality worldwide. Polypills, containing various combinations of medications for primary and secondary CVD prevention, have been developed to enhance medication adherence and reduce the healthcare burden of CVD. However, their effectiveness compared to usual care remains uncertain.

**Objective:**

This meta-analysis aimed to evaluate the effects of polypills on cardiovascular risk factors, major adverse cardiovascular events (MACE), and medication adherence.

**Methods:**

We conducted a comprehensive search for large-scale randomized controlled trials and observational studies comparing the effects of polypills *versus* usual care on CVD risk factors and events. Outcomes included changes in systolic and diastolic blood pressure (SBP, DBP), lipid profiles, occurrence of MACE, and medication adherence.

**Results:**

The use of polypills led to a statistically significant yet clinically modest reduction in SBP (mean difference -1.47 mmHg, 95% CI: -2.50 to -0.44, *p*<0.01) and DBP (mean difference-1.10 mmHg, 95% CI: -1.68 to -0.51, *p*< 0.01) compared to usual care. Polypills also showed a significant reduction in the risk of MACE (RR: 0.86, 95% CI: 0.77 -0.95, *p<*0.01). There was a non-significant reduction in LDL and HDL levels. Adherence to medication improved by up to 17% in polypill users compared to those on usual care (*p* < 0.01). A multivariable meta-regression analysis suggested that adherence may be the underlying factor responsible for the observed effect of the polypills on blood pressure.

**Conclusion:**

Polypills were found to significantly reduce SBP, DBP and MACE. An improvement in medication adherence was also observed among polypill users, which might be responsible for the significant reduction in SBP observed users. Future research might benefit from exploring a more personalized approach to the composition of polypills, which could reveal a more clinically significant impact of increased adherence on CVD outcomes.

## 1 INTRODUCTION

Cardiovascular disease (CVD) is the leading cause of mortality worldwide, with an estimated 18.6 million deaths attributed to CVD in 2019 alone [1]. The global burden of CVD has nearly doubled over the past three decades, with cases surging from 271 million to 523 million between 1990 and 2019. This continuous rise in CVD incidence and prevalence is inextricably linked to the escalating prevalence of various modifiable CVD risk factors such as hypertension and hyperlipidemia. These factors, which could primarily be prevented through the utilization of healthy lifestyle changes and pharmacotherapy, pose a significant challenge to public health [2].

Despite the availability of effective treatments and the emergence of novel therapies, adherence to recommended regimens among CVD patients remains suboptimal, leading to unfavorable short and long-term outcomes [3].

In response to the high morbidity and mortality associated with CVD, innovative strategies have been proposed to increase patient adherence to prescribed therapies. The use of polypills, which combines multiple CVD risk-reduction agents into a single tablet, represents such an approach. This treatment modality aims to enhance patient convenience and reduce polypharmacy, a frequently encountered issue among this patient population. In 2003, Wald and Law first postulated the concept of the polypill, suggesting its potential to dramatically reduce the CVD burden by over 80% [4]. Since then, polypills have undergone extensive investigation for primary and secondary CVD prevention, yielding promising results [5].

Poor adherence to medications can be attributed to many factors, including ineffective communication between patients and physicians, forgetfulness, and taking multiple pills for a long time [6]. This can result in many complications, side effects, extra costs, and failure of treatment [6]. Notably, polypills have demonstrated efficacy in improving medication adherence for secondary prevention among CVD patients. However, the consensus on the role of polypills in global CVD risk reduction remains contentious due to incomplete characterization of their safety profile and inconsistent recommendations regarding their ideal composition [3]. These findings have precipitated questions concerning the integration of polypills into standard CVD prevention strategies.

In light of this, our study sought to evaluate the effectiveness and safety of polypills in comparison to usual care. To achieve this, we performed a systematic review and meta-analysis of randomized trials and observational studies examining the use of polypills among patients diagnosed with or at risk for CVD.

## 2 METHODS

### 2.1 Systematic Literature Review

A comprehensive systematic search of PubMed, Midline, and the Cochrane Library databases was conducted following the Preferred Reporting Items for Systematic Reviews and Meta-Analyses (PRISMA) guidelines [7]. Keywords used were “polypill,” “fixed-dose,” “drug combination,” “drug combinations,” “cardio,” and “heart” combined with Boolean operators to increase search sensitivity and specificity.

### 2.2 Study Selection

Study screening was performed by two blinded reviewers (HS, MS), with disputes resolved by a third (BM). Inclusion criteria were randomized controlled trials (RCTs) and observational studies that assessed the use of standard therapy or an active medication comparator *versus* a polypill to reduce CVD, polypills a combination of antihypertensives, lipid-lowering agents and antiplatelet agents, studies including adult participants aged 18 years or older, with or without a history of previous CVD, and reported surrogate outcomes such as blood pressure or LDL level or clinical outcomes such as death from cardiovascular causes, nonfatal myocardial infarction, stroke, or the need for urgent revascularization. Non-English literature and studies with missing data were excluded. In addition, studies with placebo arms were excluded.

### 2.3 Data Extraction

The variables extracted from each study included year, prevention status, follow-up (months), study design, polypill ingredients (mg), age (years), gender, SBP/DBP mmHg, Type 2 DM, LDL-C mg/dl, BMI Kg/m^2^, adherence, and any major adverse events.

### 2.4 Result Synthesis and Meta-Analysis

Forest plots were constructed depicting the corresponding Risk Ratio (RR) and 95% confidence interval (CI) for each outcome variable. Each RR was calculated using a random-effects model, where each study was weighted by the inverse of their estimated study variance in order to account for between-study variation [8]. A multivariate meta-regression was performed to investigate the sources of heterogeneity. The covariates for this model were selected after conducting univariate meta-regressions for each study-level or participant-level characteristic variable. All variables with *P*-value < 0.05 were included in the multivariate model. The mixed-effects model was used to account for the correlation between studies. The model was fitted using maximum likelihood estimation. Publication bias was assessed using funnel plots, rank correlation tests, and Egger's regression tests [9]. All analyses were performed using R software (version 4.2.2). For cluster studies, the effective sample size was calculated using the intracluster correlation coefficient (ICC) based on the formula proposed by Donner and Klar [10]. The ICC was estimated from the available studies. The effective sample size was determined by dividing the total sample size by the design effect, calculated as 1 + (m - 1) * ICC, where m represents the average cluster size. This effective sample size was used in the meta-analysis to adjust for the clustering effect. Crossover studies were treated as parallel-group trials due to the low risk of carry-over.

### 2.5 Evaluation of Statistical Heterogeneity

Evaluation of between-study statistical heterogeneity was completed using Cochran's Q statistic and described using the associated I2 measure and *p*-values. As the Cochrane Handbook on Systematic Reviews suggested, I2 values of >30%, >50%, and >75% were used as cutoffs to indicate moderate, substantial, and considerable statistical heterogeneity, respectively [11]. Similarly, as suggested by the Cochrane Handbook, a *p*-value of <0.10 was used as a cutoff for statistically significant between/interstudy statistical heterogeneity [11]. The corresponding heterogeneity I2 value and *p*-value for readability are reported after the calculated 95% confidence interval for each respective outcome variable throughout the text.

## 3 RESULTS

### 3.1 Systematic Literature Review

**Fig. (1):**
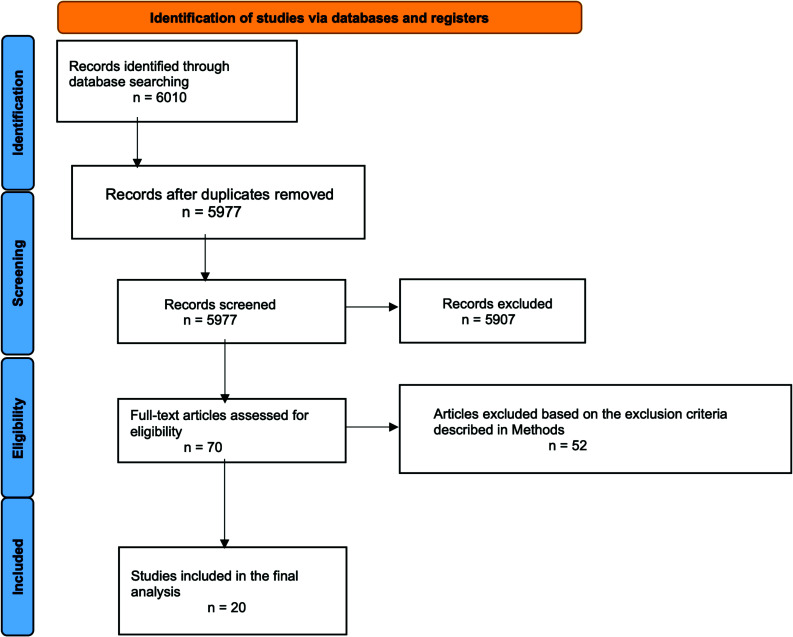
PRISMA diagram.

The search resulted in 6010 articles, of which 33 were duplicates. Of the 5977 screened articles, 70 were assessed in full-text, and 20 were included in the final analysis (Fig. **1**) [12-31].

There were 17 RCTs and three observational studies. Together, these studies included 15750 participants. Polypill was used as primary prevention in 11 studies and secondary prevention in 9 studies. Table **1** Studies with a placebo arm were excluded [32-42].

### 3.2 Quantitative Synthesis and Meta-Analysis

#### 3.2.1 Polypill Effect on Blood Pressure Compared to Usual Care

**Fig. (2):**
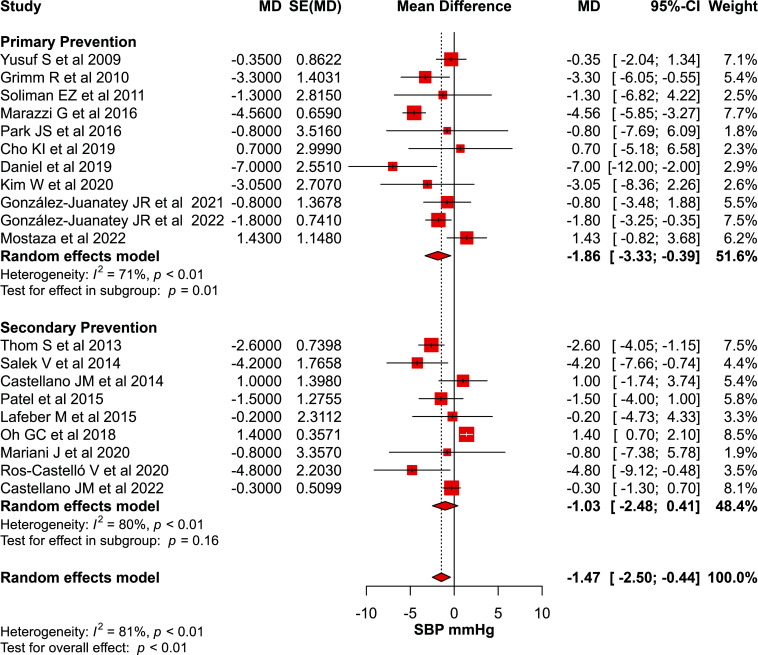
Forest plot for SBP outcome.

**Fig. (3):**
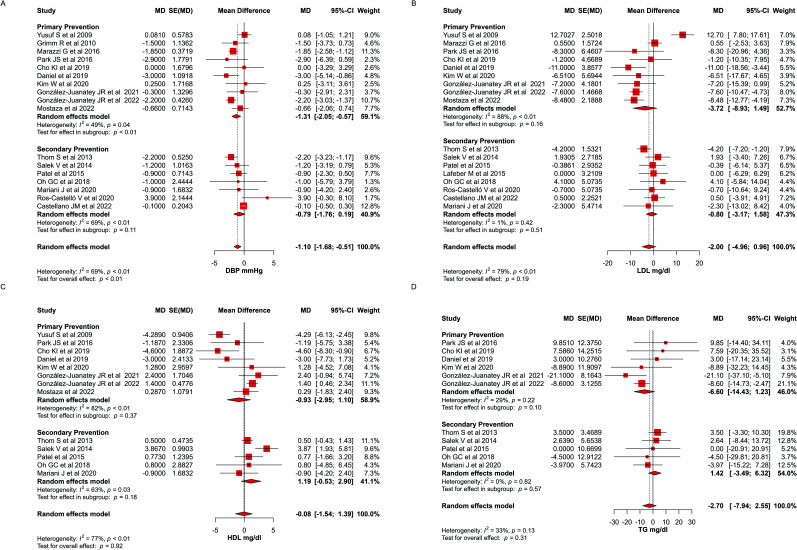
(**A-D**) Forest plot for DBP, LDL, HDL, and TG (triglyceride).

From the 20 studies that reported the effect of polypills on systolic blood pressure (SBP), there was a modest but statistically significant reduction in SBP when polypills were used as primary prevention compared to usual care (MD: -1.86; CI: -3.33 to -0.39, *p*=0.01). However, no significant differences were found when polypills were used as secondary prevention (MD: -1.03; CI: -2.48 to 0.41, *p*=0.16) (Fig. **2**). Similarly, a modest but significant reduction in DBP was observed when polypills were used as a primary prevention (MD: -1.31; CI: -2.05 to -0.57, *p*<0.01) but not when used as a secondary prevention (Fig. **3A**).

#### 3.2.2 Polypill Effect on Cholesterol Levels Compared to Usual Care

There was no significant difference in the change from baseline of low-density lipoprotein (LDL) levels between the polypill and control arms using lipid-lowering agents (MD: -2.00; CI: -4.96 to 0.96, *p*= 0.19) (Fig. **3B**). Similarly, no significant difference was noted in high-density lipoprotein (HDL) levels between the two groups (MD: -0.08; CI: -1.54 to 1.39, *p*= 0.92) (Fig. **3C**) or in triglyceride levels between the two groups (MD: -2.70; CI: -7.94 to 2.55, *p*= 0.31) (Fig. **3D**).

#### 3.2.3 Adherence

**Fig. (4):**
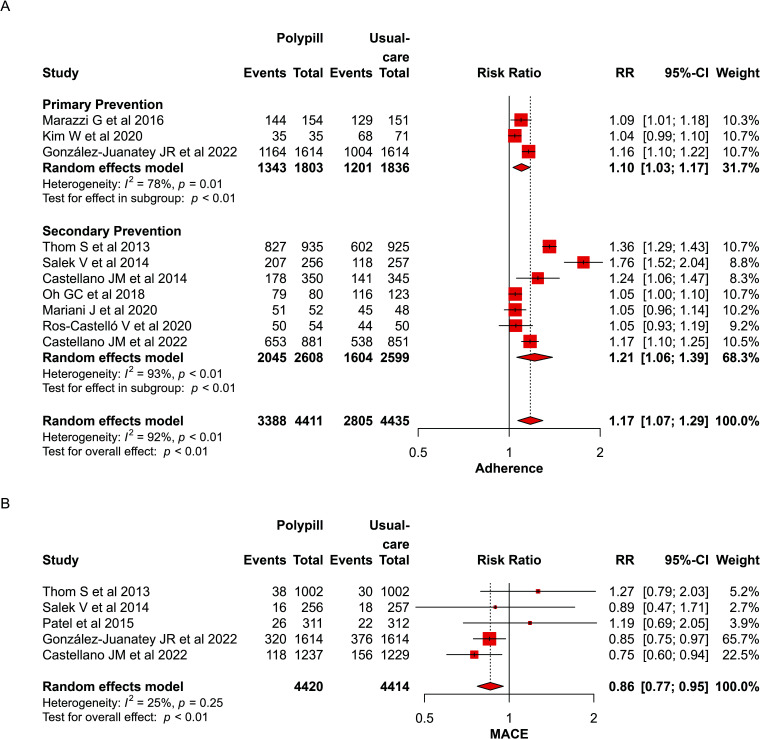
(**A** and **B**) Forest plot for adherence and MACE.

The use of polypill increased medication adherence compared to taking multiple separate pills (RR: 1.17; CI: 1.07 to 1.29, *p* <0.01) (Fig. **4A**).

#### 3.2.4 Polypill Effect on Major Adverse Cardiovascular Events (MACE)

A total of five studies included data on MACE. The use of polypill was associated with a significant reduction in MACE events (RR: 0.86; CI: 0.77 to 0.95, *p*<0.01) (Fig. **4B**).

#### 3.2.5 Investigating Heterogeneity: Meta-Regression for SBP effect size

**Fig. (5):**
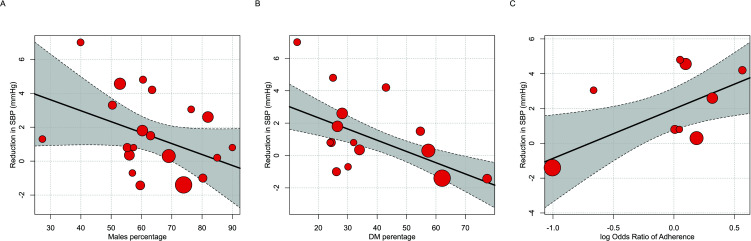
(**A-C**) Univariate meta-regression.

Based on Polypill's effect on SBP, we next analyzed the heterogeneity in our findings. I2 was high (0.81), indicating substantial variation in effect sizes across studies. To identify sources of variance, we conducted a series of univariate meta-regressions (Table **2**) to investigate the proportion of variance that could be explained by various individual- or study-level factors. Two of these variables were significantly associated with the magnitude of the SBP reduction: LogOR Adherence and Percentage of Diabetic Patients (Fig. **5**).

Then, we conducted a multivariate meta-regression to determine which variables were predictive of the SBP outcome. Two variables entered the regression model (LogOR Adherence, Percentage of Diabetic Patients) with a total R2 of 1 (Table **3**). Medication adherence showed a significant association with the reduction in SBP (SBP reduction: 1.4; CI: 0.29 to 2.5; *p* = 0.013). In addition, the Diabetic Patients Percentage was statistically significant when adjusted for LogOR Adherence (Table **3**, SBP reduction = -0.06; CI = -0.11 to -0.01; *p* = 0.02).

#### 3.2.6 Publication Bias and Sensitivity Analysis

The results of the funnel plot analysis demonstrate that the distribution of the SBP reduction is subjectively symmetrical, potentially suggesting an absence of publication bias. However, there are some concerns about the presence of publication bias since the plot reveals an asymmetry at the bottom of the graph.

**Fig. (6):**
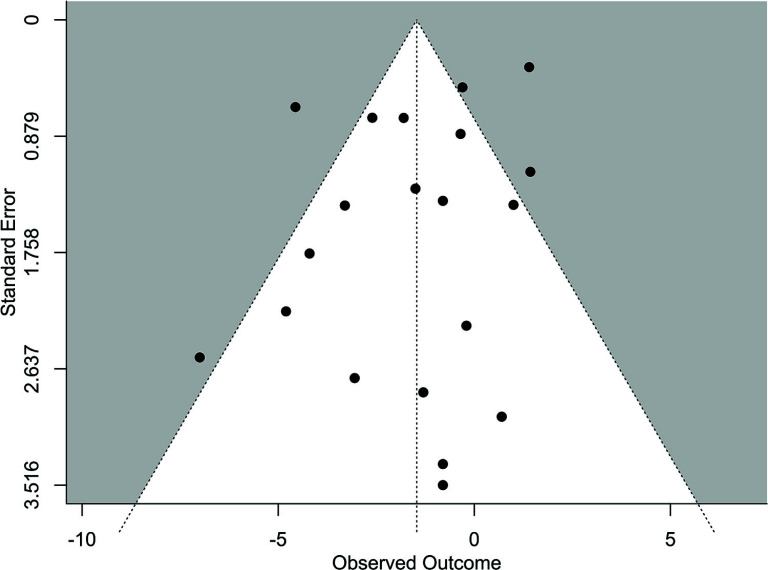
Publication bias plot.

To further evaluate the presence of publication bias, we conducted Egger's regression test, which was not statistically significant (Egger's z = -0.8781, *p* = 0.3799). We also conducted a rank correlation test for funnel plot asymmetry, and the results were also non-significant (Kendall's tau = 0.00, *p* = 1.00) (Fig. **6**). In addition, we assessed the publication bias for the rest of the variables and found no significant level of bias in any of these analyses.

Lastly, a sensitivity analysis was conducted by omitting each study at a time and seeing the observed effects on different outcomes. None of the outcomes changed except for MACE results, which became insignificant after removing the primary prevention study González-Juanatey JR *et al.* [18] or after omitting Salek *et al.* [14] or Castellano *et al.* [28] (Supplementary Fig. **S1**).

#### 3.2.7 Quality Assessment

Supplementary Fig. (**S2**) shows the risk of bias assessment. Overall, the included RCTs showed a moderate risk of bias, which was predominantly noticed in the “blinding of participants” domain, with approximately half of the studies where participants and researchers were blinded. In addition, RCTs have shown significant bias in both the “allocation concealment” and “incomplete outcome data” domains (Supplementary Figs. **S2** and **S3**). Also, it shows the risk of bias assessment for the included observational studies (Supplementary Fig. **S4**). Overall, the scales of these studies are referred to as fair quality due to the low score gained in the selection domain compared to the other two domains.

## 4 DISCUSSION

In this comprehensive meta-analysis, our evaluation of the effects of polypills compared to standard care on cardiovascular disease (CVD) risk factors and events has provided several insights. Although the use of polypills resulted in modest but statistically significant reductions in systolic and diastolic blood pressure, they did not demonstrate superior efficacy in reducing LDL and triglyceride levels or modifying HDL levels compared to standard care. Interestingly, our analysis also revealed a significant risk reduction of major adverse cardiovascular events (MACE) with polypill usage. Moreover, medication adherence improved by up to 17% among polypill users, an observation consistent with prior research [3, 43, 44].

Prior studies reported uncertain effects of polypills on CVD and mortality outcomes due to limited availability of outcomes trials and low event rates [45, 46]. However, our study found a significant reduction in MACE, SBP, and DBP. In addition, polypills were associated with increased medication adherence. These findings support the idea of the “prevention paradox,” which indicates that a slight decrease in risk across the whole population can yield more significant benefits than substantial decreases at the highest levels of risk [47-51].

Despite this, our multivariable regression analysis showed that adherence and diabetic patient percentage are responsible for the reduction observed in SBP, which counterintuitive findings observed from previous studies and meta-analyses that polypills with different components can reduce cardiovascular outcomes but rather the increased adherence associated with using it [52-54].

In addition, our study revealed that adherence was increased up to 17% with the use of polypills. This can be further increased and have more influential means for risk reduction of many risk factors using a multidisciplinary approach with recent trends using telemedicine and mobile health apps [55]. Mobile apps have proved their efficacy in reducing atherosclerotic cardiovascular diseases when compared to usual care. Moreover, these apps can also improve heart rate variability in diabetic patients compared to usual care alone. Nevertheless, the use of AI systems can detect and assess potential risk factors and the possibility of developing the disease.

This study is not without limitations. Firstly, only a few studies in the literature reported the effects of polypill on adherence, making it challenging to draw definitive conclusions on the correlation between medication adherence and clinical outcomes. Secondly, the various polypill components across different studies might have influenced the results. Furthermore, reporting of MACE was limited in the included studies, which could have influenced our analysis of the effect of polypills on cardiovascular outcomes. Lastly, significant heterogeneity was observed across the studies included in this meta-analysis.

## CONCLUSION

In conclusion, our study indicates that using polypills, compared to usual care, results in a modest reduction in blood pressure and significantly improves medication adherence. Increased adherence through polypills might be the reason for the observed reductions in SBP. Future research might benefit from exploring a more personalized approach to the composition of polypills, which could reveal a more clinically significant impact of increased adherence on CVD outcomes. Further studies with robust designs are warranted to elucidate the role of polypills in primary and secondary CVD prevention.

## Figures and Tables

**Table 1:** Demographics and baseline characteristics of included studies.

**Study, Year, Number of Patients**	**Prevention Status**	**Follow-up (mo)**	**Study Design**	**Polypill Ingredients (mg)**	**Age Years Mean (SD)**	**Male n (%)**	**SBP/DBP mmHg Mean (SD)**	**Type 2 DM n (%)**	**LDL-C mg/dl Mean (SD)**	**BMI Kg/m^2^ Mean (SD)**
Patel *et al.* 2015 623 [11]	Secondary Prevention	6-36	Clinical Trial	lisinopril 10, hydrochlorothiazide OR atenolol: 12.5 OR 50, simvastatin 40, aspirin 75	63.56 (12.60)	392 (63)	143 (20)/ 81 (12)	341 (54.7)	92.6 (37)	NR
Oh *et al.* (TELSTA-YU), 2018 203 [12]	Secondary Prevention	2	Clinical Trial	telmisartan 80, rosuvastatin 20	61.2 (10.6)	150 (73.9)	151 (12.4)/90 (9.4)	126 (62.1)	144 (28.6)	25.7 (2.8)
Selak *et al.* 2014 513 [13]	Secondary Prevention	12	Clinical Trial	lisinopril 10 with either atenolol 50 or hydrochlorothiazide 12.5, simvastatin 40, aspirin 75	62 (8)	326 (63.5%)	144(20)/83(11)	218 (43)	98.5 (31)	33 (7)
Cho *et al.* 2019 219 [14]	Primary Prevention	2	Clinical Trial	candesartan 32, rosuvastatin 20	63 (10)	125 (57)	146.6 (10)/ 85.9 (8.9)	66 (30.1%)	157.4 (28.5)	NR
Yusuf *et al.* (TIPS), 2009 2053 [15]	Primary Prevention	3	Clinical Trial	hydrochlorothiazide 12.5, ramipril 5, atenolol or nothing: 50 or nothing, simvastatin 20,aspirin 100	54 (7.9)	1152 (56)	134.4 (12)/ 85 (8)	696 (34)	116 (31)	26.3 (4.5)
González-Juanatey *et al.* (The NEPTUNO study), 2022 3228 [16]	Primary Prevention	24	Observational study	ramipril 2.5/5/10, atorvastatin 20/40, aspirin 100	63.3 (11.6)	1949 (60.3%)	140.3 (21.2)/ 81.95 (12.5)	857 (26.5)	128.5 (42.3)	28.7 (4.4)
Grimm *et al.* 2010 244 [18]	Primary Prevention	1.3	Clinical Trial	Amlodipine - 10, atorvastatin 20	56 (13.38)	123 (50.4)	132.6 (11.8)/ 81.45 (8.85)	NR	129.4 (23..2)	NR
González-Juanatey *et al.* 2021 213 [17]	Primary Prevention	1	Clinical Trial	ramipril 10, atorvastatin 40, aspirin 100	55.25 (9.7)	118 (55.3%)	N/A	52 (24.4%)	136.9 (32.8)	29.8 (3.4)
Kim *et al.* 2020 106 [19]	Primary Prevention	2	Clinical Trial	Amlodipine10, rosuvastatin20	63.09 (10.22)	81 (76.4%)	154.08 (10.16)/ 91.31 (8.26)	NR	151.12 (26.58)	26.24 (3.28)
Marazzi *et al.* (The ALL-IN-ONE trial), 2016 306 [20]	Primary Prevention	3	Clinical Trial	perindopril 10, Indapamide 2.5, amlodipine 5/10, Atorvastatin: NR	58.9 (6.03)	162 (52.9%)	143.68 (6.89)/ 91.03 (4.71)	N/A	156.96 (15.51)	24.4 (4.36)
Park *et al.* (OLSTA-D RCT), 2016 181 [21]	Primary Prevention	2	Clinical Trial	olmesartan medoxomil 40, rosuvastatin 20	61.1 (8)	104 (57.4)	150.5 (13.3)/ 92.4 (6.8)	58 (32%)	154.2 (31.4)	25.3 (2.6)
Thom *et al.* (the UMPIRE RCT), 2013 2004 [22]	Secondary Prevention	12-24	Clinical Trial	lisinopril 10, atenolol 50/hydrochlorothiazide 12.5, simvastatin 40, aspirin 75	61.85 (10.6)	1642 (81.93%)	137.35 (21.2)/ 77.75 (11.75)	564 (28.1%)	91.5 (34.3)	26.95 (4.65)
Muñoz *et al.* 2019 303 [23]	Primary Prevention	12	Clinical Trial	amlodipine 2.5, hydrochlorothiazide 12.5, losartan 25, atorvastatin 10	56 (6)	121 (39.9%)	140 (17.5)/ 83 (8)	39 (12.8)	112.6 (34.4)	30.8 (8.5)
Soliman, 2011 216 [24]	Primary Prevention	3	Clinical Trial	lisinopril 10, hydrochlorothiazide 12.5, simvastatin 20, aspirin 75	59.1 (7.2)	59 (27.3%)	165.2 (18.3)/ NR	NR	NR	NR
Mariani, 2020 100 [25]	Secondary Prevention	6	Clinical Trial	atenolol 50-100, ramipril 5-10, simvastatin 40, aspirin 100	53.7 (8.4)	90 (90%)	109.8 (13.3)/ 66.9 (9.2)	24 (24%)	124.3 (39.4)	30.1 (4)
Ros-Castelló *et al.* 2020 104 [26]	Secondary Prevention	3	Observational study	ramipril 2.5/5/10, atorvastatin 20/40, aspirin 100	69.7 (13.9)	63 (60.5%)	148.4 (17.4)/ 83.2 (13.3)	26 (25%)	105.7 (29.3)	NR
Castellano *et al.* (FOCUS), 2014 2118 [27]	Secondary Prevention	9	Observational study	ramipril 2.5/5/10, simvastatin 40, aspirin 100	64.01 (11.28)	1700 (80.26)	129.05 (18.39)/ 75.93 (10.04)	554 (26.16)	NR	27.51 (4.57)
Lafeber *et al.* 2015 78 [28]	Secondary Prevention	4-5.5	Clinical Trial	lisinopril 10, hydrochlorothiazide 12.5, simvastatin 40, aspirin 75	67 (8)	66 (85)	132 (14)/73 (9)	NR	85 (23.2)	27.5 (3.7)
Mostaza *et al.* (VULCANO), 2022 439 [29]	Primary Prevention	3.6	Clinical Trial	ramipril 2.5/5/10, atorvastatin 20/40, aspirin 100	64.7 (8.9)	262 (59.6%)	133.8 (12.4)/NR	340 (77.4%)	95.68 (30.15)	30.7 (4.9)
Castellano *et al.* (SECURE), 2022 2499 [30]	Secondary Prevention	24	Clinical Trial	ramipril 10-2.5 mg, atorvastatin 40-20 mg, aspirin 100 mg	75.9 (6.6)	1724 (69%)	129 (17.8)/74 (11.2)	1434 (57.4%)	89.2 (37.2)	27.5 (4.4)

**Table 2:** Univariate Meta-regression on SBP.

**Univariate Meta Regression on SBP Reduction**				
**Variable**	**β**	**95% CI**	***p-*value**	**R2**
Gender: Male	-0.06	-0.14,0.01	0.09	19.93
Baseline SBP	0.04	-0.07,0.16	0.46	1.79
Study Design: Observational	0.05	-2.67,2.76	0.97	0.01
Age	-0.06	-0.23,0.11	0.47	5.15
Smoker	0.04	-0.04,0.12	0.33	0.24
Follow-up in Months	0.02	-0.1,0.14	0.73	0.78
Prevention Status (Secondary Prevention)	-0.85	-2.79,1.1	0.39	9.42
Diabetic Patients Percentage	-0.07	-0.11,-0.03	0.01>	83.18
LogOR Adherence	2.84	0.47,5.21	0.02	52.49

**Table 3:** Multivariate Meta-regression on SBP reduction.

**Multivariate Meta-Regression on SBP Reduction**				
**Variable**	**β**	**95% CI**	***p-*value**	**R2**
LogOR Adherence	1.4	0.29, 2.5	0.013	100
Diabetic Patients Percentage	-0.06	-0.11, -0.01	0.02	

## Data Availability

All the data and supportive information are provided within the article.

## LIST OF ABBREVIATIONS

CVD: Cardiovascular Disease
MACE: Major Adverse Cardiovascular Events
RCTs: Randomized Controlled Trials

## STANDARDS OF REPORTING

PRISMA guidelines and methodology were followed.

PRISMA checklist is available on the publisher's website.

## SUPPLEMENTARY MATERIAL

PRISMA checklist is available as supplementary material on the publisher's website along with the published article. Supplementary material is available on the publisher's website along with the published article.
